# 

**Published:** 1877-03

**Authors:** 


### BOOKS AND PAMPHLETS RECEIVED.

A practical treatise on Diseases of the Skin. By Louis A. Duhring, M.D., etc. 1877.

Mansill's Almanac of Planetary Meteorology and New System of Science. By Richard Mansill.

The Kentucky Infirmary for Women and Children. Report.

Peripheral Paralysis. By F. T. Miles, M.D., etc. Being No. XII. of Vol. II. of "American Clinical Lectures."

Twenty-fourth Annual Announcement Medical Department University of Vermont.

Full term Extra-Uterine Gestation of the Tubo-Ovarian form, with special examination of the Sac, Uterus and Appendages. By A. Sibley Campbell, M.D.

Charter, Ordinances and By-Laws of the College of Physicians of Philadelphia.

Liebig's Extract of Malt and its Chemical Composition, Manufacture, and Therapeutical uses. By F. H. Davis, M. D.

Fourth Biennial Report of the Board of State Commissioners of Public Charities of the State of Illinois. November, 1876.

Transactions of the Medical Society of the District of Columbia. December, 1876.

Remarks on Intra-Uterine Polypi, with special reference to their Diagnosis and Surgical Treatment.

Transactions of the Wisconsin State Medical Society. 1876.

Report on the Influence of Climate on Pulmonary Diseases in Minnesota. By Franklin Staples, M.D.

---

### Medical News and Items.

Rush Medical College.—The thirty-fourth Annual Commencement Exercises of Rush Medical College were held in the new college building, corner of Wood and Harrison streets, Wednesday evening, February 21, 1877. The large amphitheatre was crowded by nearly a thousand physicians and friends of the college. Many hundreds were unable to gain admit-

### ANNOUNCEMENTS FOR THE MONTH.

**MONDAYS.** SOCIETIES.

*Mondays, March* 5 *and* 19.—Chicago Medical Society. Regular meetings.
*Mondays, March* 12 *and* 26.—Chicago Soc. of Phys. and Surgeons. Regular meetings.

CLINICS. *Every Monday.*

At Eye and Ear Infirmary, 2 P. M.—Prof. Holmes.
At Central Dispensary (Wood and Harrison sts.)—2 P.M. *Gynecological*, Dr. Adolphus; 3 P.M. *Diseases of Children*, Dr. R. S. Hall.
At Mercy Hospital—2 P. M., *Surgical*, Prof. Andrews.
At Rush College—2¼ P. M., *Medical*, Dr. Bridge.
At Chicago College, 2 P. M *Gynecological*—Prof. Merriman.

LECTURES. *Every Monday.*

At Rush Medical College (Harrison and Wood sts.)—9 to 1 o'clock, Drs. Wadsworth, Jackson, Danforth and Knox. At Chicago College—8½ to 12½, Profs. Jewell, Hyde, Hatfield and Bond; 3 to 6, Profs. Nelson and Davis, Quine and Andrews, and Roler.

**TUESDAYS.** SOCIETIES.

*Tuesday, March* 13.—Academy of Science. Regular meeting at 8 P.,M. (263 Wabashav.)

CLINICS. *Every Tuesday.*

At Eye and Ear Infirmary—2 P. M., Prof. Jones.
At County Hospital—2 P. M., *Medical*, Prof. Bevan. 3 P. M., *Surgical*, Prof. Bogue.
At Mercy Hospital—2 P. M., *Medical*, Prof. Hollister.
At Chicago College—2 P. M,, *Gynecological*, Prof. Roler.

LECTURES. *Every Tuesday.*

At Rush College—9 to 1, Drs. Owens, Bridge, Strong and Case. At Chicago College—8½ to 12½, Profs. Jewell, Quine, Merriman and Bond; [3 to 6, Profs. Johnson and Nelson, Andrews and Hatfield, and Byford.

**WEDNESDAYS.** CLINICS. *Every Wednesday.*

At County Hospital—2 P. M., *Ophthalmological*, Dr. Montgomery; 3 P. M., *Gynecological*, Prof. Fitch.
At Chicago College—3 P. M., *Gynecological*, Prof. Nelson.
At Mercy Hospital—2 P. M., *Ophthalmological*, Prof. Jones.
At Central Dispensary—2 P. M., *Surgery*, Dr. Loomis; 3, *Diseases of Chest*, Dr. Ingals; 3, *Gynecological*, Prof. Etheridge.

LECTURES. *Every Wednesday.*

At Rush College—9 to 1, Drs. Wadsworth, Ingals and Sawyer. At Chicago College—8½ to 12½, Profs. Hyde, Isham, Hatfield and Bond; 3 to 6, Profs. Davis and Curtis, Quine and Jones, and Roler.

**THURSDAYS.** CLINICS. *Every Thursday.*

At Eye and Ear Infirmary—2 P. M., Prof. Hotz.
At Mercy Hospital—2 P. M., *Medical*, Prof. Davis.
At Rush College—2 P. M., *Medical*, Prof. Ross; 3 P. M., *Diseases of the Nervous System*, Prof. Lyman.
At Chicago College—2 P. M., *Gynecological*, Prof. Merriman.
At Central Dispensary—2 P.M., *Surgical*, Dr. Graham.

LECTURES. *Every Thursday.*

At Rush College—9 to 1, Drs. Hayes, Bridge, Strong and Case. At Chicago College—8½ to 12½, Profs. Hollister, Isham, Merriman and Bond; 3 to 6, Profs. Johnson and Nelson, Andrews and Hatfield, and Byford.

**FRIDAYS.** SOCIETIES.

*Friday, March* 9.—State Microscopical Society of Illinois. Regular meeting, 8 P. M.

CLINICS. *Every Friday.*

At County Hospital—2 P. M., *Medical*, Prof. Bevan; 3 P. M., *Surgical*, Prof. Bogue.
At Mercy Hospital—2 P. M., *Medical*, Prof. Davis.
At Chicago College—2 P. M., *Gynecological*, Prof. Roler.
At Central Dispensary—2 P. M., *Diseases of Chest*, Dr. Harroun; 3 P. M., *Dermatological*, Dr. Maynard.

LECTURES. *Every Friday.*

At Rush College—9 to 1, Drs. Wadsworth, Jackson, Strong and Knox. At Chicago College—8½ to 12½, Profs. Hollister, Isham, Hatfield and Bond; 3 to 6, Profs. Davis and Curtis, Jones and Quine, and Roler.

**SATURDAYS.** CLINICS. *Every Saturday.*

At Chicago College—2 P. M., *Surgical*, Prof. Andrews or Isham; *Gynecological*, Prof. Nelson; 3 P. M., *Medical*, Prof. Johnson.
At Rush College—2 P. M., *Surgical*, Prof. Gunn.

LECTURES. *Every Saturday.*

At Rush College—9 to 1, Drs. Owens, Bridge, Sawyer and Danforth. At Chicago College, 8½ to 11½, Profs. Hollister, Quine and Hyde; 3 to 6, Profs. Nelson and Johnson, Andrews and Quine and Byford.

At all the above named Clinics visiting regular practitioners are, we believe, admitted.

At the South Side Dispensary (Chicago College) there are six daily special Clinics, for sections of the classes of the Chicago College.

The Spring Course of Rush College begins March 7th. The Winter Course of the Chicago College ends March 20th.

